# Perception of Torque is Impacted by a Subset of Features Related to the Motor Command

**DOI:** 10.1109/TOH.2023.3249473

**Published:** 2023-06-20

**Authors:** Ninghe M. Cai, Netta Gurari

**Affiliations:** 1 Ninghe M. Cai and Netta Gurari is with the Department of Physical Therapy and Human Movement Sciences, Northwestern University, 645 N Michigan Ave Suite 1100, Chicago, IL, 60611, USA; 2 Netta Gurari is with the Department of Biomedical Engineering & Mechanics, Virginia Polytechnic Institute and State University, 495 Old Turner St, Blacksburg, VA, 24060, USA

## Abstract

Accurate perception of one’s self-generated torques is integral to sensorimotor control. Here, we examined how features of the motor control task, specifically the variability, duration, muscle activation pattern, and magnitude of torque generation, relate to one’s perception of torque.

Nineteen participants generated and perceived 25% of their maximum voluntary torque (MVT) in elbow flexion while simultaneously abducting at their shoulder to 10%, 30%, or 50% of their MVT in shoulder abduction (${\mathrm{MVT}}_{\mathrm{SABD}}$). Subsequently, participants matched the elbow torque without feedback and without activating their shoulder.

The shoulder abduction magnitude affected the time to stabilize the elbow torque (p<0.001), but did not significantly impact the variability of generating the elbow torque (p=0.120) or the co-contraction between the elbow flexor and extensor muscles (p=0.265). The shoulder abduction magnitude influenced perception (p=0.001) in that the error in matching the elbow torque increased with an increased shoulder abduction torque. However, the torque matching errors neither correlated with the time to stabilize and variability in generating the elbow torque, nor the co-contraction of the elbow muscles.

These findings suggest that the total torque generated during a multi-joint task impacts the perception of a torque about a single joint; yet, effective and efficient generation of the torque about a single joint does not impact the torque percept.

## I. INTRODUCTION

Successful and seamless completion of mundane sensorimotor activities, such as opening a door, as well as advanced sensorimotor skills, such as playing tennis, require one to appropriately interact with their surroundings. These sensorimotor tasks necessitate one to not only generate desired movements, but also to accurately perceive their movements [1]. While movement generation and perception can be identified as distinct elements of sensorimotor control, these processes are not occurring independently of one another. That is, one’s generation of a movement is impacted by their perception of what is happening; and, vice versa, one’s perception of a movement is impacted by how the movement is generated [2], [3]. An important component of movement is the generation of forces about a joint, i.e. torques. In this study, we aim to provide insight into how aspects of volitional motor activation affect the torque perceptual process.

Past research mostly utilized a matching protocol to study the perception of torques [4], [5], [6], [7], [8], [9], [10], [11]. In such matching protocols, researchers investigated the connection between the generation and perception of torques using effort, which can be thought of as perceived exertion and as a correlate of motor commands. Specifically, these studies probed whether it is primarily the magnitude of torques being perceived and matched or the perceived effort [4], [5], [6], [7], [10], [11], [12], [13]. Results from these works showed that it was often the perceived effort that was matched, rather than the magnitude of torques [4], [6], [7], [14]. Hence, this literature supports the notion that the perception of one’s self-generated torques is primarily influenced by central signals related to the magnitude of the descending motor commands, underscoring the connection between the generation and perception of torques [15]. Even so, it remains to be explored whether additional aspects of the motor control process beyond torque magnitude could impact one’s perception.

Literature on optimal motor control shows that increased motor output results in greater variability in torque production [16], [17]. Additionally, an increased motor output could lead to increased stiffness, by muscle co-contraction, with the goal of stabilizing the limb [18], [19], [20]. The temporal profile of the task can also be different for increasing motor demands, as earlier studies highlight that the complexity of a motor task impacts the time required for it to be completed [21]. Since increased motor output can influence aspects of motor control, such as the variability, stabilization, and duration, it is important to understand how these elements may also influence the perception of torques.

Our previous work demonstrates that perception of an isometric flexion torque generated about the elbow is influenced by the extent to which the shoulder simultaneously abducts [11]. We expand our current work to focus not only on the changes in torque perception caused by the increase in the net torque generated, but also on changes in perception brought upon by the: i) variability of the torque generated, ii) changes in muscle activation patterns, and iii) time taken to stabilize the torques. First, we hypothesized that the shoulder abduction load impacts the variability in generating the elbow torques, activation pattern of the elbow muscles, and time to stabilize the elbow torques. Second, we hypothesized that individuals would increasingly overestimate a torque about the elbow when the torque generated about the shoulder increased, due to an increased effort of the overall task. Finally, we determined whether changes in the torque generation process corresponded to the perceptual outcomes. Combined, this study advances our current understanding of which aspects of the motor control process impact the perception of torque.

## II. METHODS

### A. Participants

This study was approved by the Northwestern University Institutional Review Board (STU00209165). Nineteen participants (ten females and nine males; mean±standard deviation age: 27±4 years) provided written informed consent prior to taking part in the study. Inclusion criteria for all participants were: 1) right-hand dominance [22], 2) no major musculoskeletal injuries to the right arm, 3) no neurological impairments, and 4) ability to understand and complete the experimental tasks.

### B. Experimental Setup

**Fig. 1.**
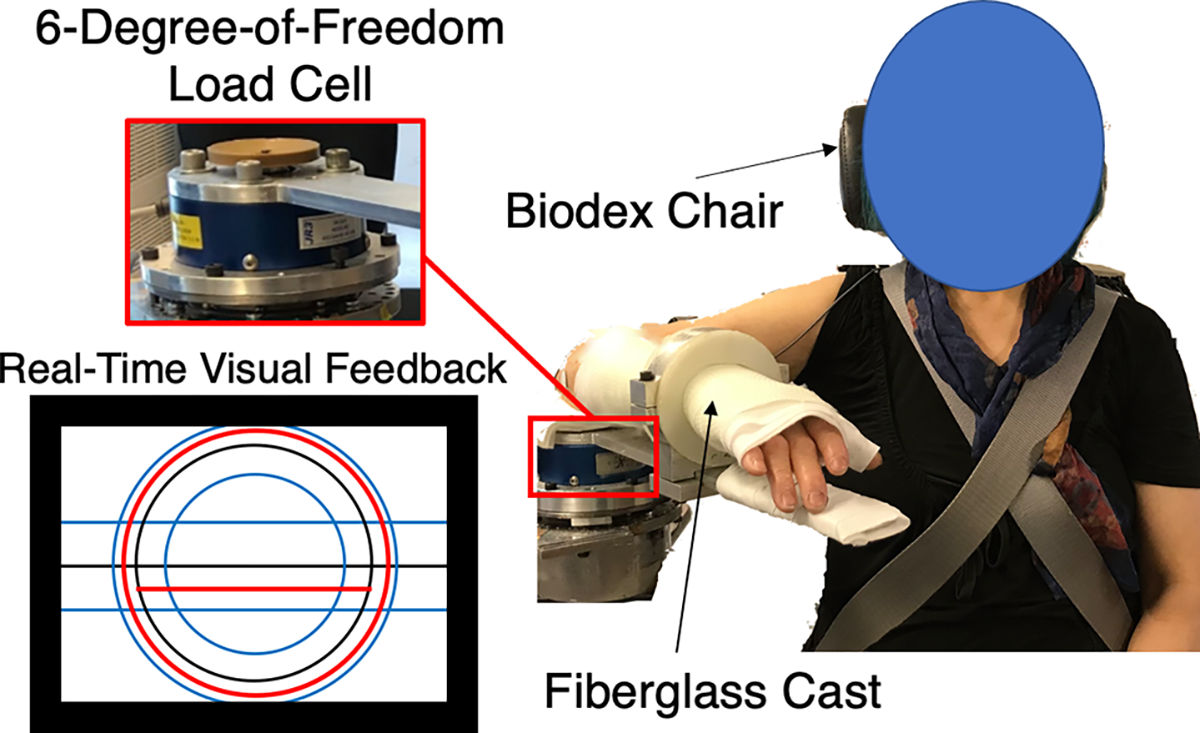
Isometric Setup. The participant’s testing arm was rigidly fixed to a six-degree-of-freedom load cell. Automated visual feedback was displayed to the participant on the monitor. The black circle and the area between the inner and outer blue circles represent the target elbow flexion torque and the acceptable range of applied elbow torques, respectively. The black horizontal line and the area between the upper and lower blue horizontal lines represent the desired shoulder abduction torque and the acceptable range of applied shoulder abduction torques, respectively. The red circle and red bar represent the participant’s self-generated elbow flexion torque and shoulder abduction torque, respectively.

Fig 1 shows the experimental setup. The participant sat in a Biodex chair (Biodex Medical Systems, Inc., Shirley, NY, United States) and their movements were restricted by seatbelts at their upper torso and waist. The participant’s testing arm was casted and affixed to a mechatronic device at angles of 85° in shoulder abduction, 45° in shoulder flexion, and 90° in elbow flexion. A six-degree-of-freedom load cell (JR3, Model: 45E15A, 1000N; Woodland, CA, USA) measured torques generated by the participant. Surface electromyography (EMG) sensors were used to obtain muscle activities at the following eight locations associated with shoulder abduction and elbow flexion: long head of the bicep brachii (BIC), lateral head of the triceps brachii (TRI), anterior deltoid (AntDel), medial deltoid (MedDel), posterior deltoid (PosDel), pectoralis major (PEC), middle trapezius (TrapM) and upper trapezius (TrapU). Specifically, activity at these muscles was recorded by two active differential surface electrodes with a 1-cm interelectrode distance (Delsys, 16-channel Bagnoli EMG System, Boston, MA, United States; 1,000×gain, 20 and 450 Hz band-pass filter). The participant received automated visual feedback from a 42-inch monitor (Panasonic TH-42PH9, Osaka, Japan) and automated auditory cues from speakers. The visual and audio information instructed the participant on how to complete the experimental protocol. The data acquisition software ran at 4 kHz, and the data were stored at 1 kHz for offline analyses.

### C. Experimental Protocol

The dominant arm of the participant was tested. We first quantified the participant’s maximum voluntary torque (MVT) in elbow flexion (${MVT}_{EF}$) and shoulder abduction (${MVT}_{SABD}$). To confirm that the participant could complete the torque matching task, the participant generated and held 25% ${MVT}_{EF}$ for four seconds while abducting at the shoulder to the desired shoulder abduction loads of 10%, 30%, and ${50\%MVT}_{SABD}$. The participant then performed three blocks of ten consecutive isometric torque matching trials, as discussed in the following section “Torque Matching Trial”; each block contained only one desired shoulder abduction load. The first two trials of each block were practice trials and excluded from data analyses. The presentation order of the blocks for each shoulder abduction load was randomized across participants using a latin-square design.

### D. Torque Matching Trial

**Fig. 2.**
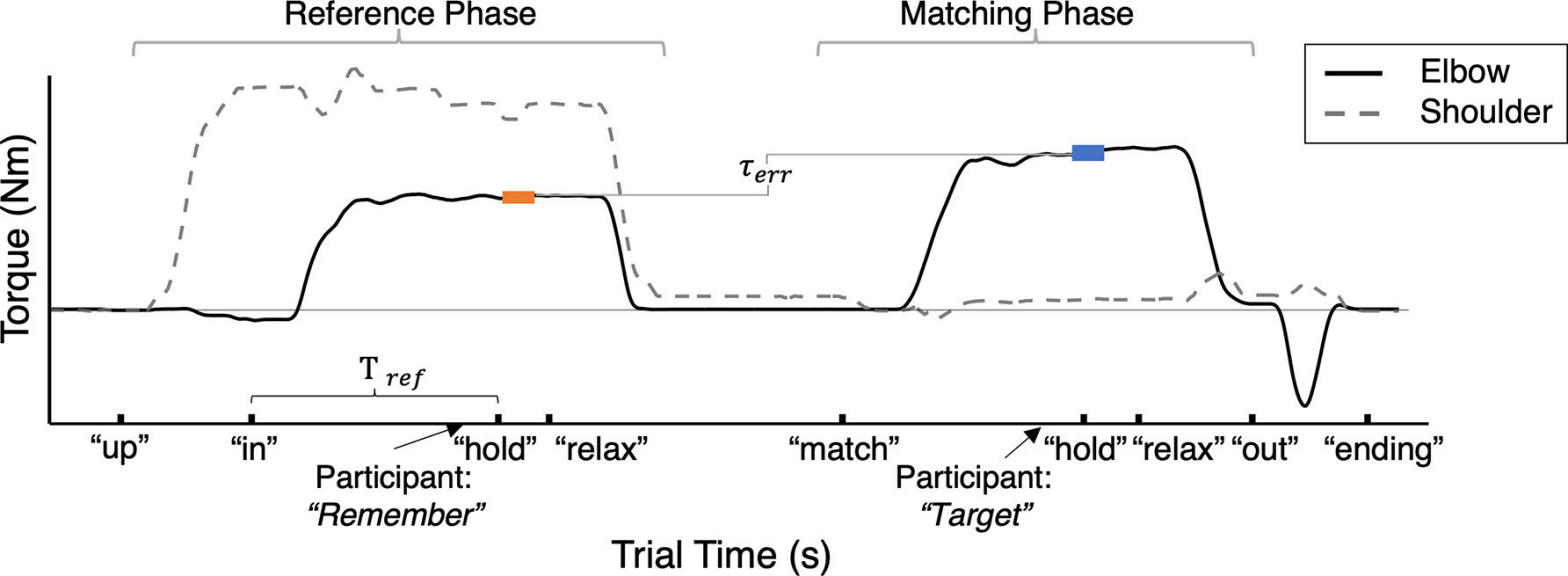
Trial Timeline and Data Segments Analyzed. Shown is an example trial timeline depicting the elbow flexion torque with a black solid line and shoulder abduction torque with a grey dashed line. Time points at which the automated audio cues played, along with the content of the cues, are indicated. The segments of data extracted to calculate the reference elbow torque, $\tau_{reference}$, and matching elbow torque, $\tau_{match}$, are identified with bold orange and blue lines respectively; the difference between $\tau_{match}$ and $\tau_{reference}$ is the $\tau_{err}$. The segment of the reference elbow torque in bold orange was also used to calculate the coefficient of variation of the elbow torque and muscle coactivation during the reference phase. $T_{ref}$ is the duration between the “in” and “relax” audio cues during the reference phase.

A torque matching trial was comprised of a reference phase in which a reference torque was generated about the elbow, followed by a match phase in which the participant aimed to generate a torque about the elbow that was equal to the reference torque without feedback; this task was performed using a single arm. This torque matching trial permitted the i) extraction of features describing the torque generation process and ii) evaluation of the torque perceived. The design of this torque matching protocol was first published in the 2021 IEEE World Haptics Conference [11]. The timeline of the trial is depicted in Fig 2 and described in detail below. Throughout the trial, the participant received automated visual and audio cues based on their actions and preset time intervals.

#### Reference Phase:

At the start of a trial, an automated audio cue “up” instructed the participant to abduct their shoulder to a desired shoulder abduction torque, $\tau_{{SABD}_{\text{des}}}$. The $\tau_{{SABD}_{des}}$ was selected to be one of three loads: 10%, 30%, or $50\%{MVT}_{SABD}$. The participant abducted at their shoulder until reaching an abduction torque within an acceptable range $\left(\tau_{{SABD}_{\text{des}}}\pm 5\%{MVT}_{SABD}\right)$. As the participant abducted about their shoulder, a red horizontal bar appeared on the screen and its height corresponded to the magnitude of the shoulder abduction torque generated. The participant knew that the desired shoulder abduction load was achieved when the red horizontal bar reached the height of a fixed black horizontal bar, which represented the desired shoulder abduction torque. The participant then maintained their generated shoulder abduction torque by keeping the red horizontal bar within the area outlined by the two fixed blue horizontal lines, which represented the acceptable range.

Subsequently, the audio cue “in” played to instruct the participant to flex about their elbow. Simultaneously, a red circle appeared on the screen, and its diameter represented in real-time the participant’s elbow flexion torque. The participant maintained the desired shoulder abduction torque and followed the visual feedback to flex about their elbow to reach the target elbow flexion torque, $\tau_{{EF}_{\text{target}}}$. The target elbow flexion torque was visually represented on the monitor by a black circle with a fixed diameter. $\tau_{{EF}_{\text{target}}}$ was 25% of the participant’s maximum voluntary torque in elbow flexion (${MVT}_{EF}$), and the acceptable target range was $\tau_{{EF}_{\text{target}}}\pm 5\%{MVT}_{EF}$. The participant generated the target elbow torque by making the red circle the same diameter as the black circle. Once achieved, the participant maintained the target elbow torque by keeping the red circle within the boundaries of the fixed inner and outer blue circles, which represent the acceptable range of the target elbow torque. The participant was required to maintain their shoulder abduction torque and elbow flexion torque within each respective acceptable range while perceiving the elbow torque. When the participant stabilized the elbow torque and was ready to hold their perceived self-generated elbow torque in memory, the participant stated aloud “remember”, which triggered the audio cue “hold” to play. The participant maintained their elbow torque for one more second, holding their perception of the torque in memory, before relaxing their entire arm.

#### Match Phase:

The participant did not receive visual feedback on their self-generated elbow torque during the match phase; that is, the red circle did not appear on the monitor. The match phase started six seconds after the reference phase with the audio cue “match”. The participant aimed to reproduce the remembered elbow flexion torque without activating their shoulder. If the shoulder torque exceeded $10\%{MVT}_{SABD}$ during the match phase, then the participant restarted the torque matching trial. When the participant believed that the previously generated reference elbow torque was reproduced, the participant stated aloud “target”, which triggered the audio cue “hold”. The participant maintained their matched elbow flexion torque for one second. Subsequently, the audio cue “relax” played, marking the end of the trial. The participant was then instructed by the audio cue “out” to quickly extend their elbow; this step was included to better relax their elbow flexors by activating the antagonist muscles [23]. There was a twenty-second break to encourage quiescent muscle activity before the beginning of the next trial.

### E. EMG Signal Preprocessing

The raw EMG data were low-pass filtered at 250 Hz and notch filtered at 60 Hz with a zero-phase shift filter. Subsequently, these data were rectified and smoothed with a 250 ms root-mean-square sliding window. The activity of the eight muscles recorded was normalized to the peak rectified and smoothed EMG obtained during the maximum voluntary contraction of each respective muscle.

### F. Data Analyses

#### 1) Quantification of Torque Generation:

For each shoulder abduction load, we quantified the following three outcomes related to the elbow torque that participants generated.

##### Stability of Torque:

We quantified the participant’s variability in maintaining the reference elbow torque using the coefficient of variation (CV). CV was defined as the standard deviation of the elbow torque, normalized to the mean, during the 0.5 s segment extracted in the reference phase after the “hold” cue (segment highlighted in orange in Fig 2). CV reflects the participant’s stability in maintaining the reference elbow torque while generating the respective shoulder abduction torque, with a greater magnitude indicating increased variability.

##### Muscle Coactivation:

The average normalized EMG activity for the elbow flexor (biceps brachii) and elbow extensor (triceps brachii) muscles was calculated for the 0.5 s-segments extracted from the reference phase of the torque matching trials (bold orange segment in Fig 2). The coactivation of the flexor and extensor elbow muscles was calculated as $\frac{{\text{normEMG}}_{\text{tricep}}}{{\text{normEMG}}_{\text{bicep}}+{\text{normEMG}}_{\text{tricep}}}\times 100\%$.

##### Duration to Stabilize Elbow Torque:

We quantified the time it took the participant to stabilize at the target elbow torque after maintaining the desired shoulder abduction torque; this duration was the time between the “in” and “hold” cues during the reference phase ($T_{ref}$, as visually depicted in Fig 2). A longer duration indicates that the participant took more time to generate the desired elbow torque during the reference phase before holding in memory the target elbow torque.

#### 2) Muscle Synergy Pattern Decomposition:

We identified the muscle synergies associated with our multi-joint shoulder abduction and elbow flexion task by analyzing the EMG data from the eight recorded muscles.

To begin, we preprocessed the EMG data. Specifically, the average normalized EMG activity for each of the eight muscles recorded was calculated for the 0.5s segments extracted from the reference phase (bold orange segment in Fig 2) and the match phase (bold blue segment in Fig 2) for each torque matching trial. The average normalized EMG activity for each of the eight muscles of each participant was integrated across torque matching conditions and phases into one matrix. The matrix for each participant consisted of the average normalized EMG activity from 8 muscles × 48 data points (two phases × eight trials × three tasks). The average normalized EMG activity of each muscle was then normalized to the corresponding maximum across all 48 data points so that each row of the matrix consisted of values ranging from 0 to 1. Finally, each row of the values in the matrix was normalized to have unit variance. The resulting matrix, *A*, was used for the muscle synergy decomposition.

To identify the muscle synergies, we used the nonnegative matrix factorization algorithm [24], [25]. For each participant, the muscle activity matrix, A, was modeled as a linear combination of time-invariant muscle synergies ($W_{1\ldots n}$), each weighted by an activation coefficient $\left(H_{1\ldots n}\right)$ that varied between phases (reference, match) and trials (1–8) of the torque matching task. This can be expressed as $A=W_{1}H_{1}+W_{2}H_{2}+\ldots +W_{n}H_{n}$. Each synergy, $W_{i}$, is a vector representing the muscle activity pattern; each element of $W_{i}$ represents a muscle’s relative contribution to this synergy. The activation coefficient, $H_{i}$, represents the relative contribution of its corresponding synergy $W_{i}$ to the overall muscle activity, A. To identify the number of muscle synergies required to reconstruct the EMGs, we increased the number of muscle synergies from one to eight. In this way, we could account for the possibility that the muscles recorded (eight) were each independent and were needed to fully explain the muscle activation patterns during the multi-joint task. We selected the minimum number of muscle synergies required to achieve a percentage of variance accounted for (VAF) of 95% [26].

To identify shared synergies across participants, we first established a threshold of similarity based on the pairwise scalar product distribution of 1,000 randomly generated synergies, i.e. the 95th percentile of the resulting 10^6^ data points. When comparing two synergies obtained from our analyses, we are able to use this threshold as a criterion to determine whether the two synergies were similar with statistical significance. The muscle synergies decomposed from an arbitrary participant was used as the reference, to which the synergies from the remaining participants were compared. Mean synergies and the activation coefficients for the group were generated by identifying similar synergies shared across participants and then averaged [27].

#### 3) Quantification of Torque Perception:

Fig 2 visually depicts the segments of data extracted for calculating the torque matching outcomes. For each trial, we extracted a 0.5 s segment of data in the reference phase when the participant indicated that the target elbow torque was remembered. We also extracted the segments of data 0.25 s before and 0.25 s after the participant indicated that the elbow torque was matched during the match phase. The average elbow torque from the 0.5 s segment extracted during the reference phase is the reference torque, $\tau_{\text{reference}}$, and that from the 0.5 s segment extracted during the match phase is the match torque, $\tau_{\text{match}}$. For each shoulder abduction load of the torque matching trials, we quantified the following outcomes related to the participant’s perceptual errors.

##### *Constant Error (*CE*):*

The CE reflects each participant’s accuracy in matching their self-generated elbow torque; it was calculated as the mean $\tau_{\text{err}}\left(\tau_{\text{match}}-\tau_{\text{reference}}\right)$ across the testing trials. A positive and negative CE indicates that the participant, on average, respectively overestimated or underestimated the reference elbow torque when matching.

##### *Variable Error (*VE*):*

The VE reflects how consistently each participant matched their self-generated elbow torque; it was calculated as the standard deviation of $\tau_{\text{err}}$ across the testing trials. A large VE indicates that the participant matched with varying elbow torques, and a VE close to zero indicates that the participant consistently matched with similar torques.

### G. Statistical Analyses

#### 1) Analysis of Torque Generation Outcomes:

To understand differences in elbow torque generation strategies among the three shoulder abduction loads, we analyzed during the reference phase the: i) coefficient of variation in maintaining the elbow torque (CV), ii) coactivation of the elbow flexor and extensor muscles, and iii) time taken to stabilize the elbow torques ($T_{ref}$). These outcome measures were fit to linear mixed-effects models. The desired shoulder abduction level, i.e. 10%, 30% or 50%, was a fixed effect and participant as a random effect [28]. For a significant main effect, we identified differences between pairs of shoulder abduction loads using post-hoc pairwise comparisons; p-values were adjusted with the Tukey method to account for the multiple comparisons.

#### 2) Analysis of Torque Perception Outcomes:

We investigated whether the accuracy (CE) and variability (VE) in matching the elbow torques significantly differed depending on the shoulder abduction load. These outcome measures were fit to linear mixed-effects models, with the desired shoulder abduction load as a fixed effect and participant a random effect [28]. For a significant main effect, we identified differences between pairs of shoulder abduction loads using post-hoc pairwise comparisons; p-values were adjusted with the Tukey method to account for the multiple comparisons.

#### 3) Correlating Torque Perception Outcomes with Torque Generation Outcomes:

We determined how changes in the motor control corresponded to perception. Specifically, we used a Spearman rank correlation to identify how, for each participant and shoulder abduction load, the elbow torque variability, muscle coactivation, and duration to stabilize corresponded to the torque matching error ($\tau_{err}$) across all testing trials.

## III. RESULTS

### A. Participant Strength

Participants’ strength was quantified by their MVTs. Across all participants, the mean±standard deviation of the strength in elbow flexion (${MVT}_{EF}$) was 48.7±18.3 Nm and in shoulder abduction (${MVT}_{SABD}$) was 54.5±24.0 Nm, respectively.

### B. Generation of Elbow Torques

#### 1) Variability in Torque Generation:

**Fig. 3.**
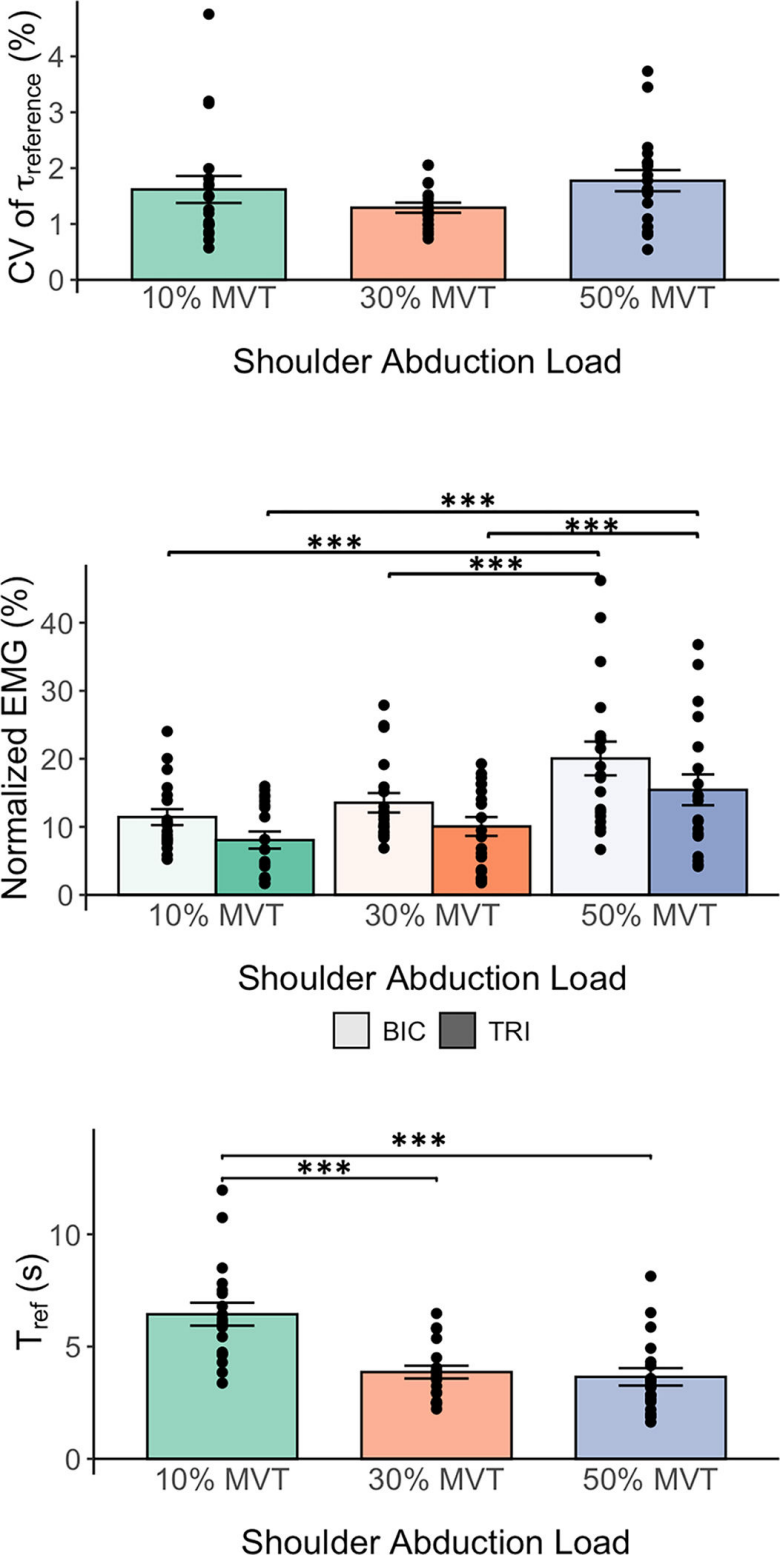
Torque Generation Outcomes. (Top) Mean and standard error of participants’ coefficient of variation (CV) for the reference elbow torque. (Middle) Mean and standard error of the participants’ normalized biceps brachii long head (BIC) and triceps brachii lateral head (TRI) activity. (Bottom) Mean and standard deviation of the time it took for participants to generate and stabilize the reference elbow torque. Each black filled circle represents data from one participant. ***: p<0.001

Participants’ variability in generating the elbow torque during the reference phase when the elbow torque was stable is summarized in Fig 3 (Top). The mean±standard deviation of the CV of the reference elbow torque when abducting to 10%, 30%, and $50\%{MVT}_{SABD}$ was 1.62±1.05%, 1.29±0.39%, and 1.77±0.83%, respectively. The CV of the reference elbow torque was not significantly affected by the shoulder abduction load (F(2,36)=2.25, p=0.120).

#### 2) Muscle Coactivation:

The normalized EMG activity of the biceps brachii long head and triceps brachii lateral head for the three shoulder abduction load conditions during the reference phase is depicted in Fig 3 (Middle). The mean±standard deviation of the normalized biceps brachii activity during the reference phase when abducting to 10%, 30%, and $50\%{MVT}_{SABD}$ was 11.43±5.11%, 13.54±6.23%, and 20.05±10.84%, respectively. The mean±standard deviation of the normalized triceps brachii activity when abducting to 10%, 30%, and $50\%{MVT}_{SABD}$ was 8.06±5.49%, 10.06±7.85%, and 15.44±9.86%, respectively. The shoulder abduction load significantly affected the muscle activation at the biceps brachii long head (F(2, 36)=15.60, p<0.001) and triceps brachii lateral head (F(2, 36)=18.50, p<0.001). The normalized EMG activity at both the biceps and triceps brachii was significantly greater when abducting to $50\%{MVT}_{SABD}$ than $30\%{MVT}_{SABD}$ or $10\%{MVT}_{SABD}$. The mean±standard deviation of the elbow muscles coactivation, when abducting to 10%, 30%, and $50\%{MVT}_{SABD}$, was 61.27±18.47%, 59.54±17.06%, and 57.41±16.71%, respectively. Coactivation of the elbow muscles was not significantly affected by the shoulder abduction load (F(2, 36)=2.57, p=0.091).

#### 3) Duration to Stabilize:

During the reference phase, after participants abducted to the desired shoulder torque of 10%, 30%, and $50\%{MVT}_{SABD}$, the time taken to stabilize at the target elbow torque, $T_{ref}$, was 6.44±2.22 s, 3.86±1.24 s, and 3.65±1.69 s, respectively (Fig 3 Bottom). $T_{ref}$ significantly differed between the three shoulder abduction loads (F(2,36)=33.99, p<0.001), being greater in the $10\%{MVT}_{SABD}$ condition than the $30\%{MVT}_{SABD}$ (p<0.001) and $50\%{MVT}_{SABD}$ (p<0.001) conditions. $T_{ref}$ did not significantly differ between the $30\%{MVT}_{SABD}$ (p<0.001) and $50\%{MVT}_{SABD}$ (p=0.841) conditions.

### C. Accuracy and Precision in Matching Elbow Torques

#### 1) Magnitude of Torques Generated:

**Fig. 4.**
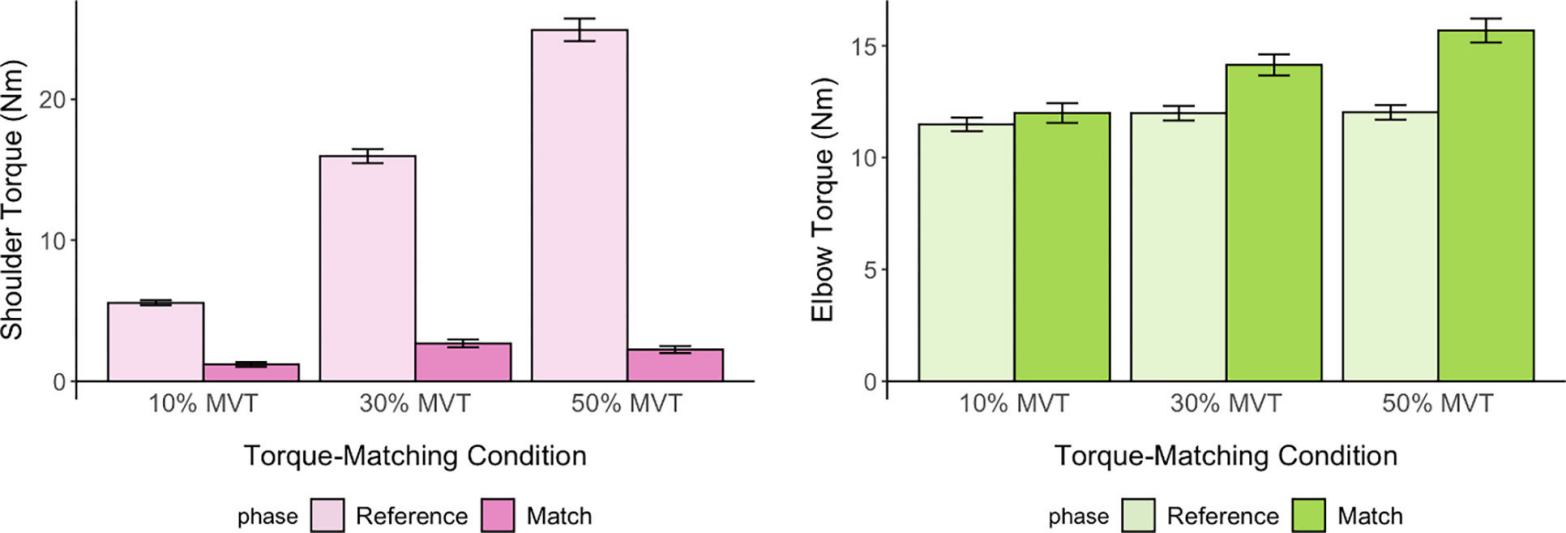
Magnitude of Torques Generated. Mean and standard error of participants’ self-generated shoulder (left) and elbow torques (right) during the reference phase and match phase.

We report the magnitude of the torques generated during the reference and match phase of the trials (Fig 4). For torque matching trials in the 10% ${MVT}_{SABD}$ condition, the shoulder abduction torque generated in the reference phase was 5.66±2.47 Nm, corresponding to 10.39±1.39% ${MVT}_{SABD}$; the reference elbow torques generated was 11.63±4.25 Nm, corresponding to 24.56±1.21% of ${MVT}_{EF}$. For trials in the 30% ${MVT}_{SABD}$ condition, the shoulder abduction torque generated in the reference phase was 15.81±7.14 Nm, corresponding to 28.78±1.96% ${MVT}_{SABD}$; the reference elbow torques generated was 11.83±4.54 Nm, corresponding to 24.56±1.03% of ${MVT}_{EF}$. For trials in the 50% ${MVT}_{SABD}$ condition, the shoulder abduction torque generated in the reference phase was 28.16±11.30 Nm, corresponding to 48.31±2.28% ${MVT}_{SABD}$; the reference elbow torques generated was 11.92±4.35 Nm, corresponding to 24.78±1.31% of ${MVT}_{EF}$. For the match phase of all trials, the shoulder abduction torque generated did not exceed 10% ${MVT}_{SABD}$ by design and as enforced with software. During the match phase in the 10% ${MVT}_{SABD}$ condition, the shoulder abduction torque generated was 1.29±1.87 Nm, corresponding to 3.02±4.33% ${MVT}_{SABD}$; the matching elbow torques generated was 11.78±5.23 Nm, corresponding to 23.87±4.43% of ${MVT}_{EF}$. During the match phase in the 30% ${MVT}_{SABD}$ condition, the shoulder abduction torque generated was 2.70±3.20 Nm, corresponding to 5.78±6.68% ${MVT}_{SABD}$; the matching elbow torques generated was 13.78±5.92 Nm, corresponding to 28.17±5.37% of ${MVT}_{EF}$. During the match phase in the 50% ${MVT}_{SABD}$ condition, the shoulder abduction torque generated was 1.85±2.70 Nm, corresponding to 4.23±6.09% ${MVT}_{SABD}$; the matching elbow torques generated was 15.81±7.14 Nm, corresponding to 32.46±8.18% of ${MVT}_{EF}$.

#### 2) Accuracy in Matching Torques:

**Fig. 5.**
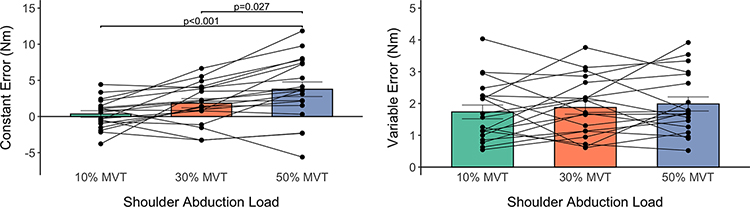
Torque Matching Outcomes. Mean and standard error of participants’ constant error (left) and variable error (right). Each line with three connected data points represents the outcome measures of one participant across the three shoulder abduction loads.

Participants’ accuracy in matching elbow torques is summarized in Fig 5 (Left). The mean±standard deviation of the CE at the 10%, 30%, and $50\%{MVT}_{SABD}$ loads was 0.34±2.02 Nm, 1.85±2.78 Nm, and 3.77±4.40 Nm, respectively. The CE was affected by the shoulder abduction load (F(2,36)=11.70, p<0.001), being greater when abducting at the shoulder to $50\%{MVT}_{SABD}$ than $10\%{MVT}_{SABD}$ (p<0.001) and $30\%{MVT}_{SABD}$ (p=0.027). The CE did not significantly differ between the $30\%{MVT}_{SABD}$ and $10\%{MVT}_{SABD}$ (p=0.102) loads.

#### 3) Variability in Matching Torques:

Participants’ variability in matching elbow torques is summarized in Fig 5 (Right). The mean±standard deviation of the VE when abducting at the shoulder to 10%, 30%, and $50\%{MVT}_{SABD}$ was 1.74±0.94 Nm, 1.87±0.92 Nm, and 1.99±0.97 Nm, respectively. The VE was not significantly affected by the shoulder abduction load (F(2, 36)=0.77, p=0.468).

### D. Muscle Activation Patterns

**Fig. 6.**
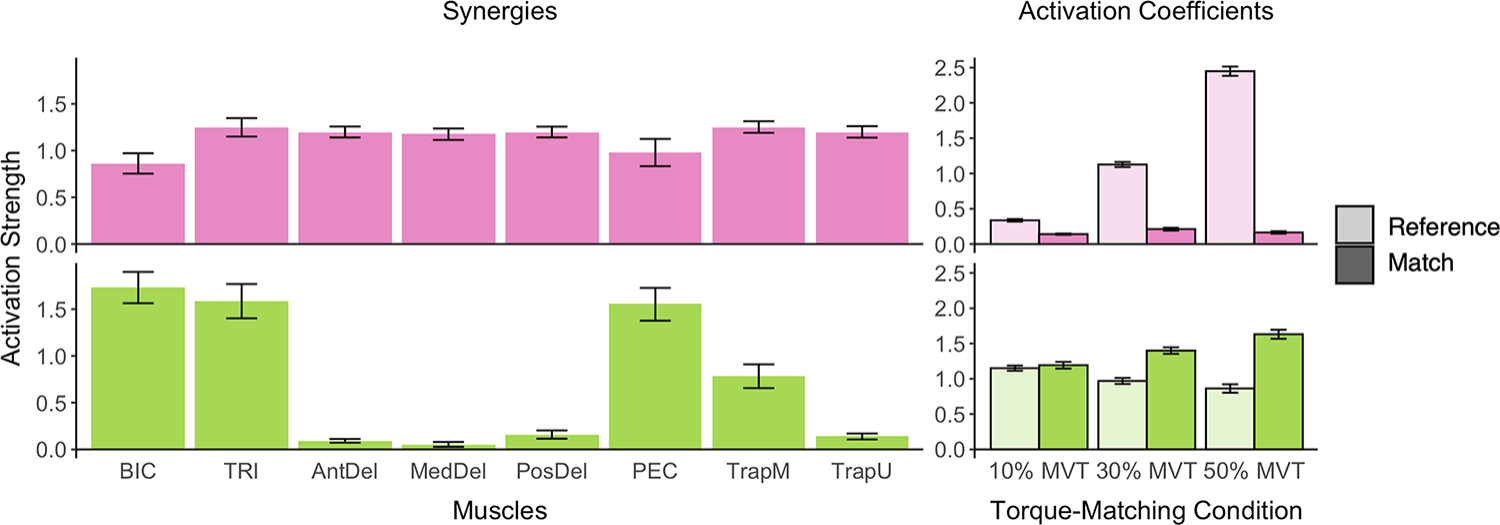
Muscle Synergies Extracted and Corresponding Activation Coefficient. Mean and standard error of muscle synergies extracted from participants during the multi-joint shoulder abduction and elbow flexion task (Left). Mean and standard error of the corresponding activation coefficients for each synergy during segments in the reference and match phases of the torque matching trial (Right).

We found that for all participants, two synergies were sufficient to explain the muscle activity recorded with 97.5±0.8% variability accounted for (VAF) and were shared by all participants. These two synergies are shown in Fig 6 (Left). The muscle synergies appear to be grouped by the isolated tasks making up the multi-joint task. One muscle synergy includes muscles primarily associated with elbow flexion and extension, corresponding to activation about the elbow. The other synergy contained muscles corresponding to shoulder abduction.

Activation coefficients of these two muscle synergies patterns during the reference and match phases are portrayed in Fig 6 (Right). The activation coefficients of these two muscle synergies provide temporal insight regarding activation, as the muscle synergies occurring during the reference phase may not be as apparent during the match phase. Results from the muscle synergy decomposition show that the activation coefficients of these two synergies during the reference and match phases follow closely what was observed from the torque profile (Fig 4). The activation coefficient of the synergy that mostly aligned with shoulder activation was greater during the reference phase and close to zero during the match phase. The activation coefficient for the synergy that mostly aligned with elbow activation had the same pattern as the net torques generated about the elbow.

### E. Correlation between Torque Generation and Perception

#### 1) Variability in Torque Generation:

We investigated whether participants’ variability in generating the elbow torque during the reference phase, when the elbow torque was stable, correlated with the torque matching error. There was not a significant correlation between the CV and $\tau_{err}$ (p>0.050) for any participant at 10% and $50\%{MVT}_{SABD}$. At $30\%{MVT}_{SABD}$, there was a strong correlation between the CV and $\tau_{err}$ for one participant (*ρ* =1, p=0.008), but no significant correlation was obtained for the remaining participants.

#### 2) Muscle Coactivation:

We investigated whether the extent to which the elbow muscles coactivated correlated with the torque matching error. For each participant at 10%, 30%, and $50\%{MVT}_{SABD}$, a significant correlation was not found between the coactivation of the biceps brachii and triceps brachii and the $\tau_{err}$ (p>0.050).

#### 3) Duration to Stabilize:

We determined whether the time taken to stabilize at the target elbow torque correlated with the torque matching error. No significant correlation was obtained for any participant at 10% and $30\%{MVT}_{SABD}$. There was a strong correlation between the $T_{ref}$ and $\tau_{err}$ for one participant at $50\%{MVT}_{SABD}$ (*ρ* =0.88, p=0.022), while no significant correlation was obtained for the remaining participants.

## IV. DISCUSSION

We presented a study that i) examined how abducting at the shoulder impacted features of the motor control at the elbow and ii) investigated how changes in the motor control features correlated with one’s perception of their self-generated elbow torque. We showed that the extent to which the shoulder abducted influenced the temporal profile of the elbow torque generation. However, the variability in maintaining the elbow torque and elbow flexor-extensor muscle coactivation patterns were similar regardless of the extent to which the shoulder abducted. Regarding accuracy in perceiving the elbow torques, we found that it was influenced by the extent to which the shoulder simultaneously abducted, yet was not correlated with the variability, muscle coactivation, and duration in generating the elbow torque. Below we expand our discussion of these findings.

### A. Torque Generation

#### 1) Control of Reference Elbow Torque:

Prior work demonstrated that an increased motor output is often accompanied by a greater presence of motor noise [16], [17], [29], [30]. In our previous study with an older-adult cohort, we saw that the CV of the reference elbow torque was significantly higher when the shoulder simultaneously abducted than when the shoulder was relaxed [10]. As a result, in our study, we hypothesized that as the shoulder abduction load increased, the generation of the elbow torque would be more variable and exhibit a higher CV. However, our current results showed that the increase in shoulder abduction load did not significantly impact the CV for the reference elbow torque in the younger population tested. That is, the young adult participants were able to maintain the reference elbow torque with similar variability among the three shoulder abduction load conditions. The age-related difference in steady force control has been implicated in previous studies, and the changes in motor unit discharge may have contributed to this difference [31], [32].

#### 2) Coactivation of Elbow Flexor and Extensor Muscles:

Prior studies suggest that with increased motor output, there is an increased stiffness at the limb due to co-contraction of the antagonist muscles [19], [20], [33], [34]. In our study, as the shoulder abduction load increased, we observed an increase in the activity of the biceps and triceps brachii. Since the biceps brachii long head is biarticular and acts on both the shoulder and elbow joint, the activity of the biceps brachii long head was expected to increase as individuals abducted their shoulder to a greater load. As the ratio in the activity of bicep and triceps brachii remained unaffected, the corresponding increase in the activity of the triceps brachii could have been to achieve stabilization during the motor task [19], [20], [33], [34].

#### 3) Temporal Profile in Generating the Reference Elbow Torque:

Researchers often design their torque matching protocols such that the participant generates the reference torque [6], [7], [35], [36], [37], [38]. Using this approach, there is the possibility that the time taken to generate and maintain the reference torque varies, which could impact perception. Even so, to the best of our knowledge research has not yet addressed whether the duration of torque generation impacts torque perception. In our study, the reference elbow torque was presented during a multi-joint task, which adds an additional layer of complexity compared to the single-joint tasks previously studied [6], [7], [35], [36], [37]. As a result, the time it took for participants to simultaneously generate the desired shoulder torque and target elbow torque is of interest. We found that the time participants took to stabilize the reference elbow torque differed among the shoulder abduction load conditions. While participants did not have difficulty generating a small shoulder abduction load, namely, 10% of the participants’ maximum voluntary shoulder abduction torque, they spent significantly more time coordinating their shoulder torque with the target elbow flexion torque before stabilizing. One explanation for the increased amount of time required to stabilize may be as follows. The long head of the biceps brachii is a biarticular muscle, contributing primarily to elbow flexion, but also acting on the shoulder. Given the biomechanical coupling of the elbow and shoulder joints, flexing about the elbow could impact the torque generated about the shoulder. Hence, this biomechanical coupling might make it difficult to generate and control a relatively small shoulder abduction load at the shoulder when the elbow flexes to a moderate magnitude of torque.

#### 4) Muscle Activation Pattern:

Our EMG decomposition results demonstrate that the muscle synergies and their corresponding activation coefficients decomposed from our multi-joint task reflect the orthogonal actions and the corresponding magnitudes of the net torques generated. As such, we did not find that the muscle synergy decomposition provides additional insightful information regarding the motor activation process of our task. This result could be because the torques generated in our task are relatively simple and isometric. Furthermore, by design, our torque matching task enforced three distinct levels of shoulder abduction during the reference phase and required individuals to relax at the shoulder during the match phase. As a result, the muscle activation pattern highly correlated with the torque constraints we placed on the task.

### B. Torque Perception

Research indicates that perception of a self-generated torque is largely informed by signals related to the descending motor commands [5], [39], [40], [41]. In our previous work, we found that, in older adults, an elbow torque was overestimated if perceived simultaneously with a shoulder abduction torque [10]. Results from this current study in young adults show a similar trend. As individuals increasingly abducted at the shoulder while perceiving their self-generated elbow torque, they increasingly overestimated the magnitude of the torque that they generated about their elbow. Abducting at the shoulder likely influenced their perception about the elbow, suggesting that the perception of self-generated torques are not independent to each joint but influenced by the overall motor commands of the task. This finding on the individuals’ representation of torque perception echos the classic theories on the hierarchy of motor control [42], [43], [44]; the perception of torque is likely organized not at the level of the individual joints, but at a higher level. Results from our current study in young adults provide additional evidence as to the role of central signals on the perception of torque. However, as discussed above, there are other motor control features involved in the generation of elbow torques, which may also vary from the change in shoulder abduction loads. In turn, these altered motor control features may additionally modulate the perception of torque. Below we discuss how these changed motor control features correlated with our torque perception outcomes.

#### 1) Variability in Torque Generation:

A previous study demonstrated that an increased variability in the generation of the reference torque could negatively affect the precision in perception [45]. During our torque matching task, individuals generated the reference elbow torque while simultaneously abducting at the shoulder. The long head of the biceps brachii is a biarticular muscle that is used for both shoulder abduction and elbow flexion. As the shoulder abduction levels differed across conditions, we expected the variability of the reference elbow torque to be impacted by the shoulder activation [29], [30], [46]. In our study, however, we did not observe an increase in the variability of the reference elbow torque generated among the three shoulder abduction loads. Additionally, the VE in matching the reference elbow torque did not significantly differ among the three shoulder abduction loads. Furthermore, analyses did not reveal a correlation between the variability of the reference elbow torque generated and the torque matching error. Therefore, the impact of variability in torque generation on torque perception was not apparent in our current study.

#### 2) Muscle Coactivation:

For a given net torque about a joint, an increase in the activation of agonist and antagonist muscles acting about a joint would mean a greater amount of total motor unit activation. In our current work, we observed an increase in the activity of both the biceps brachii long head and triceps brachii lateral head, but not the ratio between them, across the shoulder abduction loads. The increase in the activation of the biceps and triceps brachii corresponded with the increase in shoulder abduction load and with the degree of overestimation of the self-generated elbow torque. This aligns with our hypothesis that the magnitude of a self-generated torque would be perceived based on the motor activation required for the task.

There was not a change in the extent to which the elbow flexor and extensor muscles coactivated across the shoulder abduction loads. We also did not see a correlation between the level of muscle coactivation and the torque matching error. As a result, we are unable to conclude whether the muscle coactivation about the elbow contributed to the perception of the reference elbow torque.

#### 3) Torque Generation Duration:

In perceiving joint position using a similar memory-based matching protocol, researchers found that the presentation time of the target position affected participant accuracy and variability [47]. Given this discovery, it is possible that the perception of a torque may also be impacted by the duration in which participants generate the reference torque. In our study, all participants maintained the reference elbow torque for one second. However, the time it took for participants to reach and stabilize at the target elbow torque with simultaneous shoulder abduction varied. Despite the difference in the temporal profile, the torque matching error was not found to correlate with the time it took for participants to stabilize at the reference torque.

Findings from our current work corroborate the idea that torque perception is influenced by centrally generated signals related to the descending motor commands. As the intensity of the overall motor commands increased due to the increase in shoulder abduction load, the torque generated about the elbow was increasingly overestimated. As a result, we conclude that the perception of self-generated torques is influenced by changes in the descending motor commands.

### C. Significance and Future Directions

This study provides further evidence on the role of descending motor commands in the perception of a self-generated torque. We showed that the error in accurately perceiving an elbow torque during a task involving the shoulder is driven by the overall motor demands of the task. Only dominant arms of participants were tested in this study. As arm dominance can affect variability in motor control [48], [49], future work can explore potential differences in the influence of motor commands on the perception of torques between arms. To further characterize the perception of torques during a multi-joint task, studies in the future can extend the current matching task design to other scenarios involving modulation of various joints. For example, future work could investigate perception about the shoulder joint with varying elbow torque magnitudes, perception about the elbow joint with varying elbow torque magnitudes, and perception about the elbow joint when joints that are less biomechanically and neurologically coupled are activated.
